# Efficacy and safety of indobufen- versus aspirin-based dual antiplatelet therapy following percutaneous coronary intervention: a systematic review and meta-analysis

**DOI:** 10.1097/MS9.0000000000005205

**Published:** 2026-06-09

**Authors:** Savaira Khalid, Khushal Khan, Muhammad Haris, Khushnood Khan, Muhammad Qataada Khan, Muhammad Hamza Abdullah Khan, Khizar Ahmad, Intikhab Alam, Sikander Ahmed Zahid, Khyam Aziz, Sikandar Ali, Jafar Ronaq, Mohammad Ebad Ur Rehman, Muhammad Sheraz Hameed, Rahmat Gul Omarzai

**Affiliations:** a Department of Medicine, Lady Reading Hospital, Peshawar, Pakistan; b Department of Medicine, Khyber Medical College, Peshawar, Pakistan; c Department of Medicine, Khyber Teaching Hospital, Peshawar, Pakistan; d Department of Medicine, Ayub Teaching Hospital, Abbottabad, Pakistan; e Department of Medicine, Saidu Group of Teaching Hospitals, Swat, Pakistan; f Department of Medicine, Rawalpindi Medical University, Rawalpindi, Pakistan; g Department of Medicine, Nangarhar Medical University, Jalalabad, Afghanistan

**Keywords:** aspirin, dual antiplatelet therapy, indobufen, percutaneous coronary intervention

## Abstract

**Background:**

Indobufen is a potential alternative to aspirin in dual antiplatelet therapy (DAPT) regimens after percutaneous coronary intervention (PCI) due to its superior safety profile and comparable efficacy. This systematic review and meta-analysis compares the efficacy and safety of indobufen-based DAPT versus aspirin-based DAPT following PCI.

**Methods:**

A systematic literature search was conducted on PubMed, Embase, Cochrane, and ClinicalTrials.gov using MeSH terms and keywords related to “Percutaneous Coronary Intervention,” “Platelet Aggregation Inhibitors,” and “Aspirin,” from inception to March 2026. Randomized controlled trials (RCTs) and cohort studies assessing indobufen post-PCI were included. A random-effects meta-analysis was performed using the Mantel–Haenszel method in RevMan to pool risk ratios (RR) and 95% confidence intervals (CIs).

**Results:**

Six studies comprising 13 044 patients were included. Indobufen use significantly reduced Bleeding Academic Research Consortium (BARC) type 2 bleeding (RR = 0.57, 95% CI 0.33–0.97), BARC type 2, 3, and 5 bleeding (RR = 0.63, 95% CI 0.45–0.90), and gastrointestinal adverse events (RR = 0.64, 95% CI 0.51–0.80). No significant differences were observed between both groups for cardiac death (RR = 1.23, 95% CI 0.72–2.12), myocardial infarction (RR = 0.92, 95% CI 0.52–1.64), ischemic stroke (RR = 0.90, 95% CI 0.58–1.39), stent thrombosis (RR = 1.12, 95% CI 0.45–2.82), and BARC type 3 or 5 bleeding (RR = 0.83, 95% CI 0.57–1.22).

**Conclusion:**

Indobufen demonstrates comparable antiplatelet efficacy to aspirin while improving safety. Further large-scale, high-quality RCTs are warranted to solidify evidence and inform future guidelines, particularly to assess the role of indobufen in patients at high bleeding risk.

## Introduction

Percutaneous coronary intervention (PCI) is a minimally invasive non-surgical procedure aimed at alleviating stenosed or occluded coronary arteries, thereby restoring vital blood flow to the ischemic myocardial tissue[1]. PCI is a cornerstone in the management of patients with coronary artery disease (CAD), which is the third leading cause of death worldwide, contributing to approximately 17.8 million fatalities each year[2]. Dual antiplatelet therapy (DAPT), which according to the recent guidelines typically consists of aspirin and clopidogrel, is the standard of care to prevent thrombotic complications that occur post-PCI. These complications include mainly stent thrombosis, recurrent myocardial infarction (MI), ischemic stroke, and death[3]. Clopidogrel irreversibly inhibits the P2Y12 receptor, blocking glycoprotein IIb/IIIa activation and reducing platelet aggregation[4]. Aspirin exerts its antiplatelet effect by irreversibly inhibiting the active site of cyclooxygenase-1 (COX-1). COX-1 is essential for the synthesis of thromboxane A2, a potent inducer of platelet aggregation[5]. Even at low doses, long-term use of aspirin is linked to a higher incidence of adverse effects. These include gastrointestinal (GI) discomfort and a significant risk of bleeding, prompting interest in alternative antiplatelet strategies[6]. Recent American and European guidelines endorse alternative approaches. These include monotherapy with P2Y12 inhibitors after a brief course of DAPT in select number of patients, in order to reduce the risk of bleeding^[^7,8^]^. However, strong evidence indicates that the efficacy of monotherapy post-PCI is optimal only when P2Y12 inhibitors that are more potent than clopidogrel are used, such as ticagrelor[9]. The efficacy of these potent antithrombotic agents (particularly ticagrelor) as monotherapy rivals that of DAPT. However, they also carry a significantly high risk of major bleeding, which remains a major concern[10].

HIGHLIGHTS

- Six studies comprising 13 044 patients were included in this meta-analysis.
- Indobufen significantly reduced Bleeding Academic Research Consortium type 2, 3, and 5 bleeding, as well as gastrointestinal (GI) adverse events, compared to aspirin.
- Indobufen and aspirin were comparable in terms of cardiac death, myocardial infarction, ischemic stroke, and stent thrombosis.
- Indobufen demonstrates a favorable bleeding and GI safety profile.

Indobufen is a drug with a mechanism of action similar to aspirin but with a key distinction: its inhibition of COX-1 is reversible. As a result, platelet function is restored within 24 hours. Additionally, it has minimal impact on prostacyclin[11]. Therefore, indobufen is associated with a significantly lower risk of bleeding and GI side effects while maintaining its efficacy as an antiplatelet drug[12]. Emerging evidence suggests that indobufen, when combined with clopidogrel, could provide a similar antithrombotic effect compared to the traditional aspirin-plus-clopidogrel regimen, with a potentially improved safety profile[13]. Although some findings suggest comparable efficacy in the post-PCI population, the evidence remains inconclusive.

A previous meta-analysis that utilized the same intervention and control groups included a more generalized population, including adults and pediatric patients with any coronary heart disease[14]. Another recent meta-analysis compared indobufen with aspirin but assessed patients with stroke in addition to patients with CAD[13]. An updated meta-analysis pooling the latest available evidence on this topic is crucial. Therefore, we undertook a systematic review and meta-analysis comparing the efficacy and safety of indobufen-based DAPT with aspirin-based DAPT in patients who have undergone PCI.

## Materials and methods

This meta-analysis was conducted in accordance with the Cochrane Handbook for Systematic Reviews of Interventions[15] and reported following the Preferred Reporting Items for Systematic Reviews and Meta-Analyses (PRISMA) guidelines[16]. Our study protocol is registered with the International Prospective Register of Systematic Reviews (PROSPERO). Artificial intelligence (AI) was not utilized at any point in this meta-analysis, as reported using the TITAN guidelines[17].

### Data sources and search strategy

We performed a comprehensive electronic search across the Cochrane Central Register of Controlled Trials, MEDLINE, Embase, and ClinicalTrials.gov from their inception until March 2026. Additionally, we screened reference lists of included studies and relevant systematic reviews to identify further studies. Conference proceedings were utilized to search for gray literature. Medical Subject Headings (MeSH) and synonyms for (“Percutaneous Coronary Intervention”) AND (“Platelet Aggregation Inhibitors” OR “Indobufen” OR “Dual Antiplatelet Therapy”) AND (“Aspirin”) were used. The detailed search strategy is reported in Supplemental Digital Content Table S1, available at: http://links.lww.com/MS9/B232.

### Eligibility criteria

We included randomized controlled trials (RCTs) and cohort studies comparing indobufen plus a P2Y12 receptor inhibitor with the standard regimen of aspirin plus a P2Y12 receptor inhibitor in post-PCI patients. Studies were excluded if they had a design other than RCTs, prospective cohort studies, or retrospective cohort studies, such as single-arm studies, case–control studies, cross-sectional studies, and animal studies. Only articles published in English were included.

### Study selection and data extraction

Duplicate removal and initial screening were conducted using Rayyan[18]. Two independent reviewers screened the titles and abstracts, excluding irrelevant studies. Full-text screening was then performed based on eligibility criteria. Disagreements were resolved by a third reviewer. Data were extracted into a pre-piloted Excel spreadsheet, including author name, publication year, country, sample size, study design, mean age, gender distribution, hypertension, diabetes, smoking, previous MI, previous stroke, indobufen regimen, aspirin regimen, and outcomes. When available, adjusted effect estimates from multivariable analyses were preferentially extracted and pooled.

### Outcomes

The primary outcome was cardiac death. Secondary outcomes included MI, ischemic stroke, stent thrombosis, bleeding, all-cause mortality, and GI adverse events.

### Risk of bias assessment

The risk of bias in RCTs was evaluated using the Cochrane Risk of Bias 2.0 tool (RoB 2.0)[19].

This tool assesses bias in five domains, which comprise: (1) bias caused by the randomization process; (2) bias due to deviations from intended interventions; (3) bias arising from missing outcome data; (4) bias in the measurement of the outcome; and (5) bias in the selection of the reported result. For observational studies, the Newcastle–Ottawa Scale (NOS) was used, assessing the selection of study groups, their comparability, and the ascertainment of exposure or outcome[20]. Two independent reviewers performed the assessments, categorizing studies as having high, low, or some concern of bias. Any disagreements were settled by a third investigator.

### Data synthesis

All meta-analyses were conducted using Review Manager (RevMan, version 5.4.1)[21]. For dichotomous outcomes, the Mantel–Haenszel method was applied, and risk ratios (RRs) with 95% confidence intervals (CIs) were extracted. A random-effects model was used for meta-analyses. Heterogeneity was assessed using Higgins’ *I*^2^ statistic, and results were presented as forest plots. All analyses were stratified on the basis of study design. Leave-one-out sensitivity analyses were undertaken.

### Certainty of evidence assessment

The Grades of Recommendation, Assessment, Development, and Evaluation (GRADE) framework was utilized to assess the certainty of evidence. Two authors independently assessed the certainty of evidence; disagreements were settled by a third author.

## Results

### Search results

**Figure 1.**
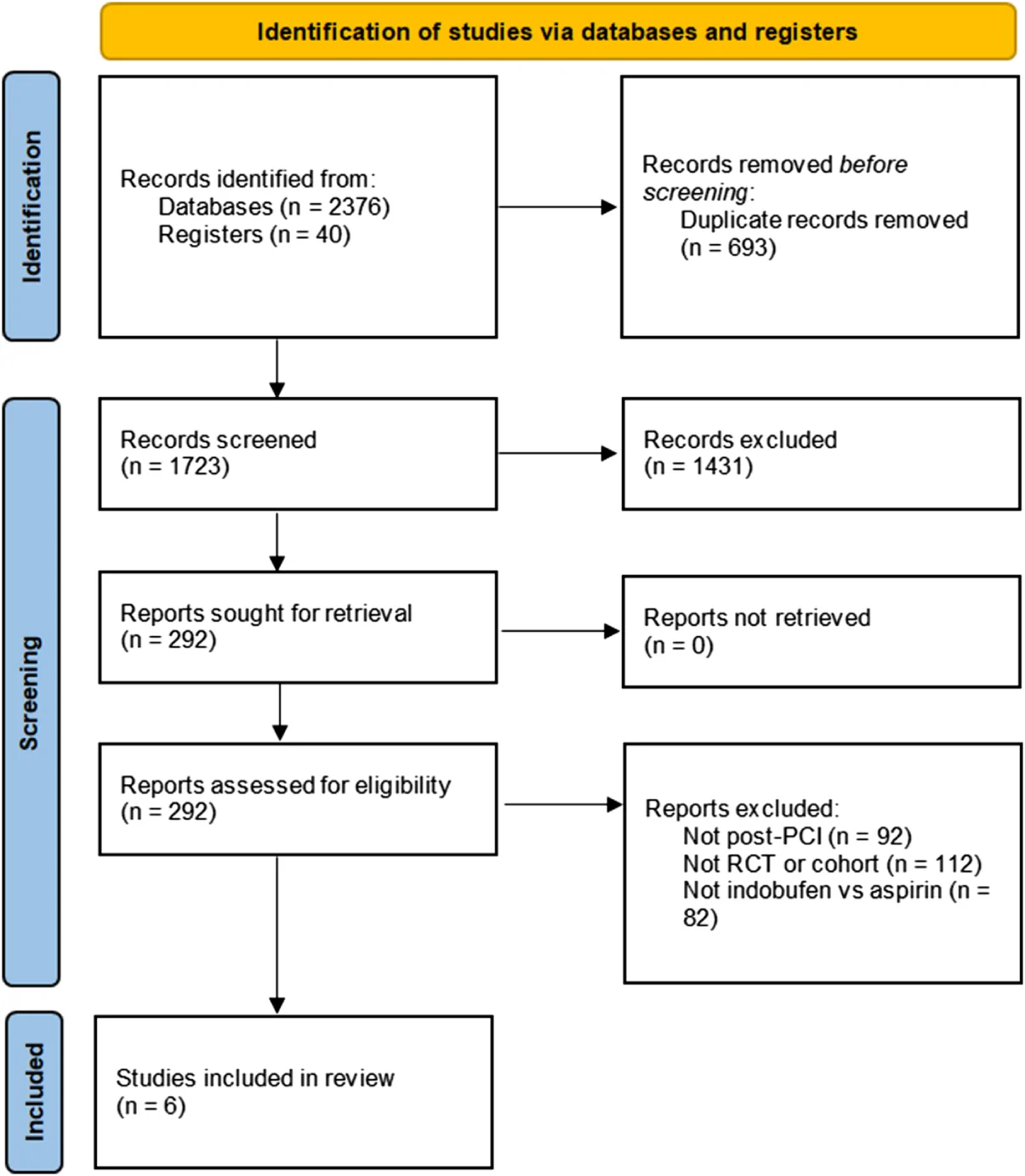
PRISMA flowchart.

Our initial search yielded a total of 2416 articles. After removing duplicates, 1723 articles underwent title and abstract screening. Afterward, 292 articles were retrieved for full-text screening, and six articles were found to meet our inclusion criteria and were included in the meta-analysis. Details of the study selection process are shown in Figure 1.

### Study characteristics

Out of the six included studies, three were retrospective cohorts, and three were RCTs^[^22–27^]^. All six studies were conducted in China from 2022 to 2025, potentially limiting the external validity of our findings. The total study population was 13 044 patients. There were a total of 3573 patients in the indobufen group and 9471 patients in the aspirin group. The characteristics of the included studies are summarized in Table 1.

**Table 1:** Characteristics of included studies.

Study ID	Country	Study design	Sample size	Mean age, in years (SD)	Male (%)	HTN (%)	Diabetes (%)	Smoking (%)	Previous MI (%)	Previous stroke (%)	Indobufen regimen (dose, frequency, duration)	Aspirin regimen (dose, frequency, duration)	Median follow-up (IQR)	Funding
Dai C. 2024[26]	China	RC	7135(689 vs 6446)	63.5 (10.5)	77.9	64.72	32.7	19.34	16.2	6.37	Indobufen 100 mg BD plus P2Y12 receptor antagonist for 6–12 months	Aspirin 100 mg od plus P2Y12 receptor antagonist for 6–12 months	1 year	National Program on Key Basic Research Project of China; National Natural Science Foundation of China; Shanghai Clinical Research Center for Interventional Medicine; Shanghai Shenkang Key Clinical Research Project; Shanghai Science and Technology Committee; Key Medical and Health Projects of Xiamen Province; Zhongshan Hospital Affiliated to Fudan University
Dai W. 2025[25]	China	RC	612 (306 vs 306)	75.7 (NR)	48.9	77.6	35.6	27.7	11.4	18.0	Indobufen 100 mg BD plus clopidogrel 75 mg OD for 12 months	Aspirin 100 mg OD plus clopidogrel 75 mg OD for 12 months	1 year	National Natural Science Foundation of China, Science and Technology Project of Tianjin Municipal Health Committee, Tianjin Medical University, National Science and Technology Innovation 2030
Jiang 2025[22]	China	RCT	240 (120 vs 120)	68.5 (12.2) vs 69.7 (10.2)	54.6	55	19.6	NR	NR	NR	Indobufen 100 mg BD plus clopidogrel 75 mg OD for 12 months	Aspirin 100 mg OD plus clopidogrel 75 mg OD for 12 months	1 year	Not specified
Liu 2026[27]	China	RC	255 (90 vs 165)	64.46 (10.41) vs 63.42 (9.39)	72.9	65.1	33.7	26.7	29.0	18.8	Indobufen 100 mg BD plus clopidogrel 75 mg OD for 12 months/ticagrelor 90 mg BD for 12 months	Aspirin 100 mg OD plus clopidogrel 75 mg OD for 12 months/ticagrelor 90 mg BD for 12 months	1 year	Program for National Science Funds of China
Wu 2023[24]	China	RCT	4551 (2258 vs 2293)	61.1 (8.3)	65.2	67.2	34.5	24.7	6	5.6	Indobufen 100 mg BD plus clopidogrel 75 mg OD for 12 months	Aspirin 100 mg OD plus clopidogrel 75 mg OD for 12 months	1 year	Not specified
Wu 2024[23]	China	RCT	251(110 vs 141)	73.9(5.6) vs 73.0(5.5)	75.69	67.33	30.67	35.85	NR	11.15	Indobufen 100 mg BD plus clopidogrel 75 mg OD for 12 months	Aspirin 100 mg OD plus clopidogrel 75 mg OD for 12 months	1 year	Clinical Research Fund Project of Zhejiang Medical Association; Guiding Scientific and Technological Project of Quzhou

RC, restrospective cohort; RCT, randomized control trial; SD, standard deviation; HTN, hypertension; MI, myocardial infarction; NR, not reported; BD, twice a day; OD, once a day; IQR, interquartile range

### Risk of bias in included studies

All studies were thoroughly assessed for risk of bias via RoB 2.0 and NOS for RCTs and cohorts, respectively. For the cohorts, the NOS score ranged from 7 to 9, indicating a low risk of bias. All RCTs were at a low risk of bias. The summary of risk of bias assessments is depicted in Supplemental Digital Content Figure S1, available at: http://links.lww.com/MS9/B231, and Supplemental Digital Content Table S2, available at: http://links.lww.com/MS9/B233.

### Meta-analysis of primary outcome: cardiac death

**Figure 2.**
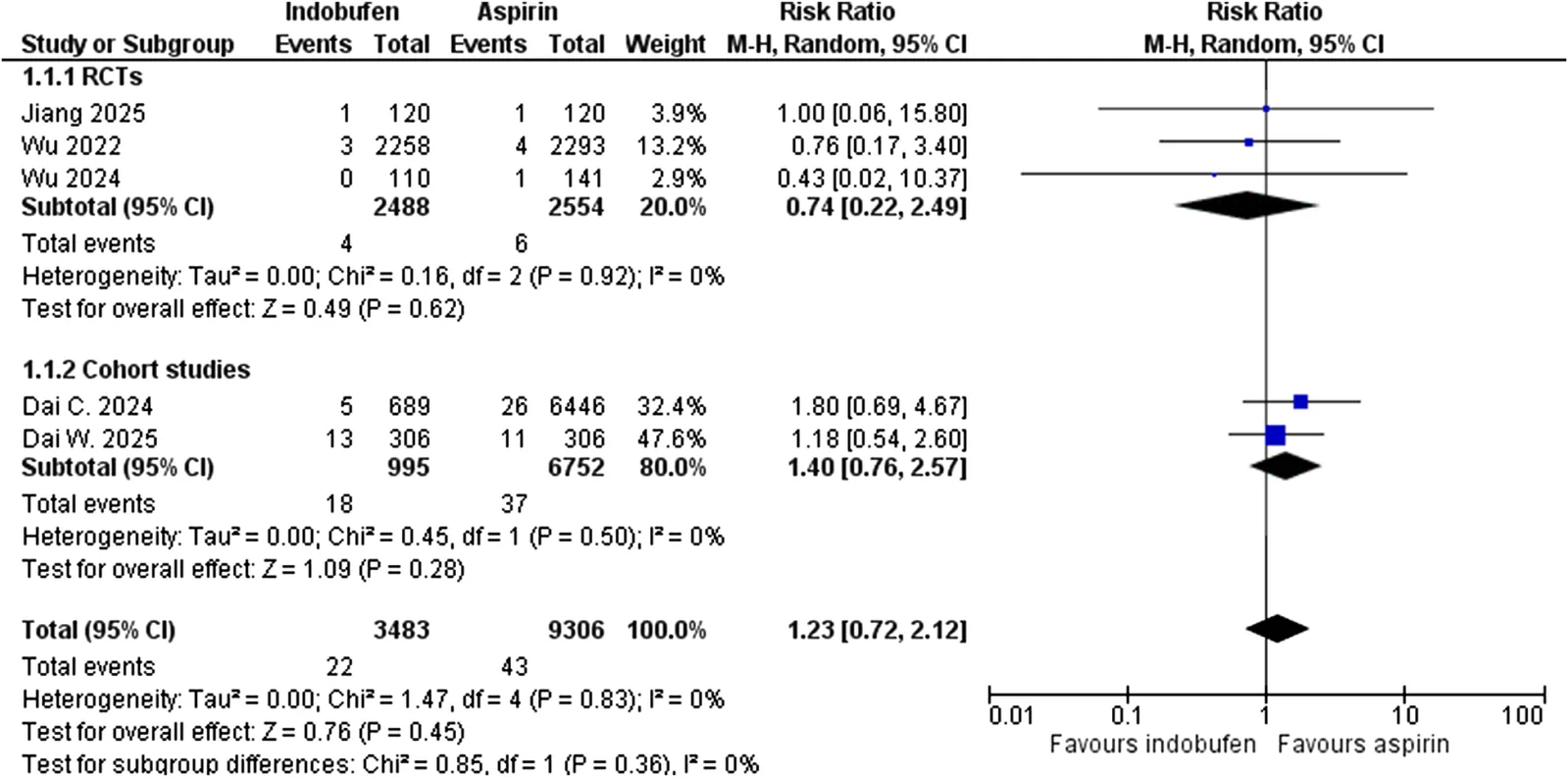
Forest plot of cardiac death.

Five studies reported cardiac death, including 12 789 patients (indobufen: 3483 vs aspirin: 9306). Statistical analysis revealed no significant difference between the two groups in terms of cardiac death (RR 1.23, 95% CI 0.72–2.12; *P*-value = 0.45; *I*^2^ = 0%), as shown in Figure 2. Results of subgroup analysis on the basis of study design as well as leave-one-out sensitivity analyses demonstrated similar findings. The overall certainty of evidence was evaluated to be moderate for RCTs and very low for cohort studies (Supplemental Digital Content Table S3, available at: http://links.lww.com/MS9/B234).

### Meta-analysis of secondary outcomes

#### Myocardial infarction

Four studies comprising a total of 12 538 patients (indobufen: 3373 vs aspirin: 9165) reported MI. Upon analysis, no significant difference was found between the two groups (RR = 0.92, 95% CI 0.52–1.64; *P*-value = 0.79; *I*^2^ = 0%), as depicted in Supplemental Digital Content Figure S2, available at: http://links.lww.com/MS9/B231. Results of subgroup analysis based on study design, as well as leave-one-out sensitivity analyses, demonstrated similar findings. The overall certainty of evidence was evaluated to be moderate for RCTs and very low for cohort studies (Supplemental Digital Content Table S3, available at: http://links.lww.com/MS9/B234).

#### Ischemic stroke

Four studies with a total of 12 549 patients (indobufen: 3363 vs aspirin: 9186) were included. Statistical analysis revealed no significant difference between the group receiving indobufen and the aspirin group (RR 0.90, 95% CI 0.58–1.39; *P*-value = 0.41; *I*^2^ = 0%). The meta-analysis is depicted in Supplemental Digital Content Figure S3, available at: http://links.lww.com/MS9/B231. Results of subgroup analysis based on study design, as well as leave-one-out sensitivity analyses, demonstrated similar findings. The overall certainty of evidence was evaluated to be moderate for RCTs and very low for cohort studies (Supplemental Digital Content Table S3, available at: http://links.lww.com/MS9/B234).

#### Stent thrombosis

Three studies in our meta-analysis reported the incidence of stent thrombosis among study participants, resulting in a total sample size of 11 937 patients (indobufen: 3057 vs aspirin: 8880). No significant difference between the two groups was reported (RR 1.12, 95% CI 0.45–2.82; *P*-value = 0.80; *I*^2^ = 0%), as reflected in Supplemental Digital Content Figure S4, available at: http://links.lww.com/MS9/B231. Results of subgroup analysis based on study design, as well as leave-one-out sensitivity analyses, demonstrated similar findings. The overall certainty of evidence was evaluated to be moderate for RCTs and very low for cohort studies (Supplemental Digital Content Table S3, available at: http://links.lww.com/MS9/B234).

#### Bleeding Academic Research Consortium (BARC) bleeding

Three studies in our meta-analysis recorded the occurrence of Bleeding Academic Research Consortium (BARC) type 2, 3, or 5 bleeding. The risk of bleeding was significantly reduced with indobufen-based DAPT (RR = 0.63, 95% CI 0.45–0.90; *P*-value = 0.01; *I*^2^ = 50%) (Supplemental Digital Content Figure S5, available at: http://links.lww.com/MS9/B231). The overall certainty of evidence was evaluated to be high for RCTs and low for cohort studies (Supplemental Digital Content Table S3, available at: http://links.lww.com/MS9/B234). When only BARC type 2 was bleeding considered, indobufen had a significantly lower risk of bleeding (RR = 0.57, 95% CI 0.33–0.97; *P*-value = 0.04; *I*^2^ = 64%) (Supplemental Digital Content Figure S6, available at: http://links.lww.com/MS9/B231). The overall certainty of evidence was evaluated to be high for RCTs and very low for cohort studies (Supplemental Digital Content Table S3, available at: http://links.lww.com/MS9/B234). No difference was observed when only BARC type 3 or 5 bleeding was considered (Supplemental Digital Content Figure S7, available at: http://links.lww.com/MS9/B231). The overall certainty of evidence was evaluated to be moderate for RCTs and very low for cohort studies (Supplemental Digital Content Table S3, available at: http://links.lww.com/MS9/B234). Results of subgroup analysis based on study design, as well as leave-one-out sensitivity analyses, demonstrated similar findings.

#### GI adverse events

Four studies in our analysis reported the occurrence of adverse GI events with a total of 8253 patients (indobufen: 1195 vs aspirin: 7058). Indobufen-based DAPT was associated with a significantly reduced risk of GI adverse events (RR = 0.64, 95% CI 0.51–0.80; *P*-value < 0.0001; *I*^2^ = 0%). This is depicted in Supplemental Digital Content Figure S8, available at: http://links.lww.com/MS9/B231. Results of subgroup analysis on the basis of study design as well as leave-one-out sensitivity analyses demonstrated similar findings. The overall certainty of evidence was evaluated to be moderate for RCTs and low for cohort studies.

## Discussion

Our meta-analysis, which included six studies with 13 044 patients, provides valuable insights into the comparative safety and efficacy of indobufen and aspirin in post-PCI patients. While no significant differences were observed in ischemic outcomes – namely cardiac death, MI, ischemic stroke, or stent thrombosis – indobufen demonstrated a significantly lower risk of major bleeding (BARC type 2, 3, or 5 bleeding) and GI adverse events.

Indobufen shows no significant difference compared to aspirin in preventing mortality due to cardiovascular causes in our meta-analysis. Cavalcante *et al* reported similar rates of major cardiovascular events between indobufen and aspirin groups, in addition to other clinical outcomes[13]. A recently published analysis in 2024 by Zhang *et al*, comparing indobufen and aspirin, reported similar findings in terms of cardiac events, including death from a cardiac cause, recurrence of angina pectoris, and incidence of MI, emphasizing the comparable effectiveness of indobufen with aspirin[14]. These results can be attributed to the similar mechanism of action of both drugs via inhibition of the COX-1 enzyme within platelets. Indobufen inhibits platelet aggregation in a manner comparable to aspirin by reducing the production of platelet thromboxane through reversible inhibition of COX-1[28]. It is noteworthy that indobufen is already approved in China and Europe for treating atherosclerotic cardiovascular disease but is not currently approved for use in the United States[29].

Comparable outcomes were also noted in ischemic stroke rates, as evidenced by the OPTION trial (hazar ratio [HR]: 0.96, *P* = 0.91)[24]. These findings confirm that indobufen is non-inferior to aspirin in preventing thrombotic complications post-PCI, a crucial factor for its clinical viability. However, various RCTs assessing the effect of indobufen in preventing ischemic stroke show contrasting results. Liu *et al* suggested that indobufen combined with clopidogrel is more effective and safer than aspirin combined with clopidogrel in patients with minor stroke or high-risk TIA[30]. However, Pan *et al* reported findings that do not support the use of indobufen for secondary stroke prevention in patients with moderate-to-severe ischemic stroke[31]. Although these studies were conducted in populations distinct from post-PCI patients, they provide insight into the cerebrovascular efficacy of indobufen across different thrombotic settings. This contextual evidence supports its potential role in mitigating ischemic stroke risk, a clinically relevant complication in the post-PCI period, while not implying direct equivalence between stroke-specific and PCI populations.

No significant differences were observed in MI or stent thrombosis rates. Several prior studies have assessed major adverse cardiovascular and cerebrovascular events (MACCE), a composite outcome that includes endpoints such as cardiac death, MI, ischemic stroke, and stent thrombosis. Dai *et al*[25] and Wu *et al*[23] both reported no significant differences in MACCE, further supporting our findings that indobufen provides non-inferior ischemic protection compared to aspirin.

Our analysis demonstrates a reduced risk of bleeding following the use of indobufen compared to conventional aspirin-based anticoagulation in post-PCI patients. All our included studies reported bleeding in terms of the BARC scale. Cavalcante *et al* reported similar findings, suggesting that indobufen exhibits an antithrombotic effect comparable to that of aspirin with lower risk of bleeding occurrences compared to aspirin at 1-year follow up[13]. Zhang *et al* in 2024 reaffirmed that indobufen significantly reduces the incidence of bleeding events[14]. In patients with multivessel coronary disease, indobufen-based DAPT offers protection from ischemic events while minimizing the risk of bleeding events compared to aspirin-based DAPT at 1-year follow-up post-PCI[32]. All these findings further strengthen our results in highlighting the superior function of indobufen in terms of bleeding complications following coronary intervention and can likely be attributed to the reversible inhibition of COX-1 compared to irreversible inhibition by aspirin. Therefore, indobufen inhibits platelet aggregation in a manner comparable to aspirin but the antiplatelet effect of indobufen diminishes faster, leading to a relatively low risk of bleeding[28]. Indobufen’s reversibility within 24 hours may provide a distinct advantage for patients requiring urgent surgical interventions, where long-acting antiplatelet agents pose challenges. This property could make indobufen particularly appealing in the perioperative management of high-risk cardiovascular patients, though further studies are needed to explore this application[33]. Another key implication is the potential role of indobufen in high-bleeding-risk populations. Current guidelines recommend shortened DAPT durations (1–3 months) for patients at high risk of bleeding, followed by antiplatelet monotherapy^[^34,35^]^. Given its lower bleeding risk compared to aspirin, indobufen could be a promising alternative in these settings. However, its role within contemporary de-escalation strategies remains unclear and warrants further research.

The use of aspirin has historically been associated with an increased occurrence of GI symptoms, such as acute gastritis and gastric ulcers. Our meta-analysis reveals that indobufen, as antiplatelet therapy following PCI, has better GI outcomes and therefore can be used as an alternative to aspirin in cases of aspirin-intolerance or adverse gastric events. Zhang *et al* also reported fewer GI reactions with indobufen compared to aspirin[14].

A major strength of our meta-analysis is the minimal heterogeneity across key outcomes (*I*^2^ = 0% for cardiac death, MI, stroke, and stent thrombosis). Most of the outcomes were reported consistently throughout the included studies. Additionally, all included studies adhered to proper DAPT protocols, minimizing confounding from inconsistent antiplatelet use. However, certain limitations must be acknowledged. First, all included studies were conducted exclusively in China, which should be emphasized when interpreting our findings. Differences in patient demographics, genetic polymorphisms affecting platelet reactivity, background cardiovascular risk profiles, and clinical practice patterns may limit the external applicability of these results to non-Chinese and global populations. As such, caution is warranted when extrapolating these findings to broader, ethnically diverse cohorts. Second, while our analysis incorporated both RCTs and high-quality retrospective studies to maximize available evidence, combining different study designs may introduce some variability in effect estimates. Observational studies, in particular, are more susceptible to selection bias and residual confounding, which could influence the magnitude or direction of outcomes, despite stratification of results on the basis of study design. Third, follow-up durations in most included studies were short to moderate, which limits the ability to draw conclusions regarding the long-term efficacy and safety of indobufen. Consequently, the durability of its antithrombotic effect, as well as the incidence of late adverse events such as bleeding, recurrent ischemic events, or stent thrombosis over extended periods, remains uncertain and warrants further investigation in studies with longer-term follow-up. Fourth, the effect of comorbidities such as hypertension and diabetes could not be assessed in detailed subgroup analyses due to the limited number of included studies. Fifth, none of the included studies clarified how GI adverse events were defined, potentially introducing heterogeneity to our results. Sixth, publication bias could not be assessed due to the limited number of studies. Seventh, despite most of the available evidence on the topic hailing from China, no Chinese database was utilized for the literature search.

## Conclusion

Indobufen-based antiplatelet therapy appears to be a potential alternative to conventional aspirin-based DAPT post-PCI. Current evidence suggests it may provide comparable cardiovascular and cerebrovascular outcomes, with a possibly lower risk of bleeding and GI complications. However, these findings are based on a limited number of studies, and further research is needed to confirm its efficacy and safety. Future investigations should include large-scale, multicenter RCTs in diverse populations to evaluate the generalizability of indobufen’s effects. Comparisons with newer P2Y12 inhibitor monotherapy regimens, such as ticagrelor, could clarify its role within contemporary antiplatelet strategies. Longer-term follow-up studies are also warranted to assess whether the potential safety benefits persist and how they may influence long-term cardiovascular outcomes. Additionally, pharmacoeconomic analyses may help determine its cost-effectiveness relative to aspirin, particularly in populations requiring prolonged antiplatelet therapy.

## Data Availability

The datasets will be provided by the corresponding author upon reasonable request.

## Research registration unique identifying number (UIN)

CRD420250654198.
